# Oxcarbazepine induced systemic lupus erythematosus: a clinical challenge^☆^

**DOI:** 10.1016/j.abd.2025.501154

**Published:** 2025-06-28

**Authors:** Eduardo López Vera, Elisabeth Gómez Moyano, María Ayala Blanca, María Salas Cassinello

**Affiliations:** a Department of Dermatology, Hospital Regional Universitario de Málaga, Málaga, Spain; b Department of Pathology, Hospital Regional Universitario de Málaga, Málaga, Spain; c Department of Alergy, Hospital Regional Universitario de Málaga, Málaga, Spain

Dear Editor,

Systemic lupus erythematosus triggered by medications is a serious adverse effect that is often incorrectly identified within the range of erythema multiforme diseases, posing significant diagnostic challenges.

A 46-year-old man was referred because of an erythematous, targetoid, and coalescent rash of acute onset over the last 48 hours. Patient history included elevated blood pressure and borderline psychiatric disorder under treatment with hydrochlorothiazide and oxcarbazepine for over two years. Recently, paracetamol and metamizol were added because of EBV infection a month prior. On physical examination, confluent erythematous violaceus and targetoid lesions affected the face with a “butterfly wing” distribution, as well as the oral mucosa, neck, and upper trunk. Denudation was intermittently observed (Figure 1, Figure 2). Joint tenderness and stiffness were also present.

**Figure 1:**
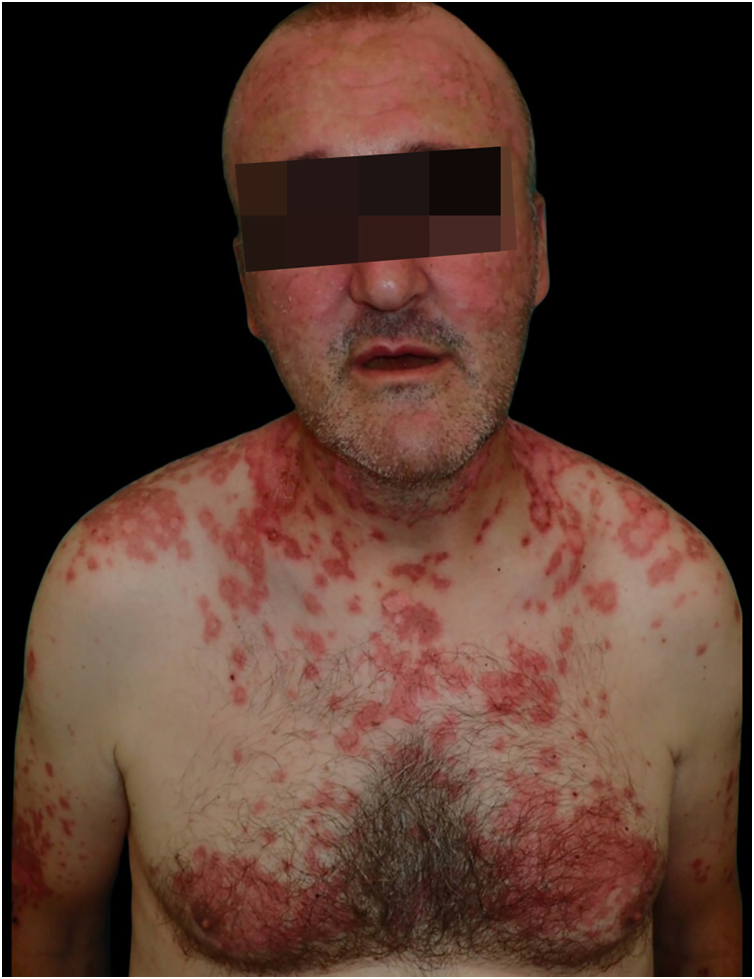
Erythematous targetoid coalescent rash with cephalocaudal distribution and central erosion involving the face, neck, upper trunk, and back.

**Figure 2:**
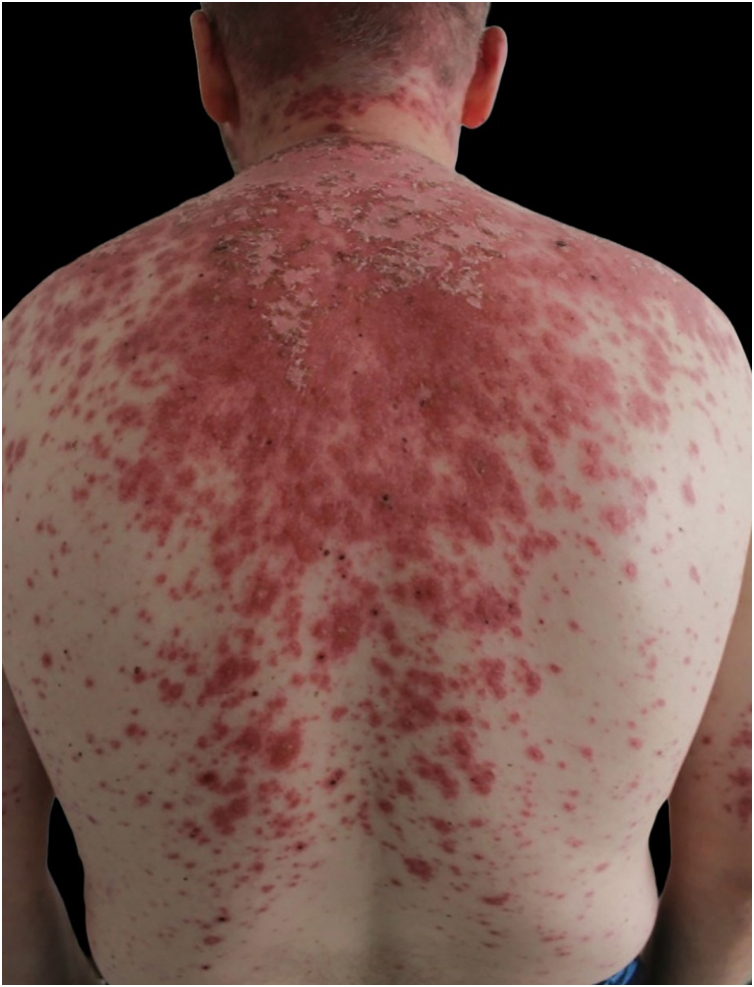
Erythematous coalescent rash with central erosion in upper trunk.

Skin biopsy revealed vacuolar degeneration of the basal layer, necrotic keratinocytes, and a superficial and perivascular lymphocytic and polymorphonuclear inflammatory infiltrate (Fig. 3). Concurrently, Direct Immunofluorescence (DIF) demonstrated granular deposits of IgM and C3 at the dermo-epidermal junction. Other DIF markers (IgG, IgA and fibrinogen) resulted in negative.

**Figure 3:**
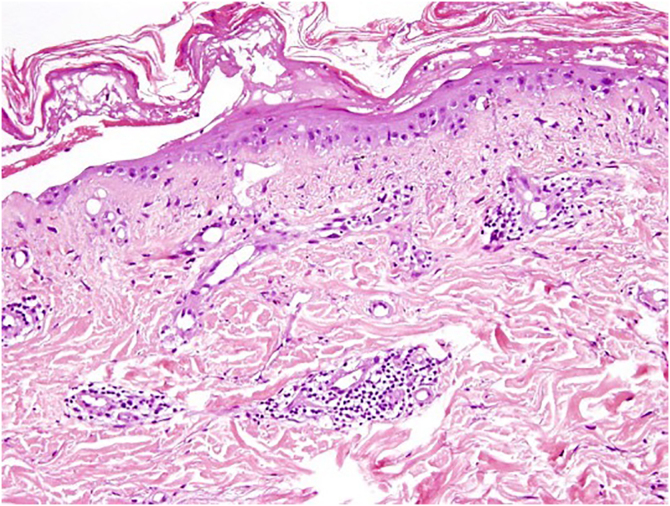
Histological findings showing focal epidermal necrosis and vacuolar degeneration of the basal layer, with lymphomonocytic and neutrophilic perivascular and upper-dermal infiltrates (Hematoxylin & eosin, ×100).

Blood tests showed thrombocytopenia (110 × 10^9^/L), lymphopenia (0.5 × 10^9^/L), positive nuclear antibodies at 1/40 tittle with a homogeneous pattern, and low C3 levels (70). Proteinuria was absent.

Oxcarbazepine and metamizole were withdrawn, and treatment with intravenous methylprednisolone 60 mg daily in descending regime was initiated, with complete resolution of skin lesions and hematological and immunological parameters. Subsequently, a Lymphocyte Transformation Test (LTT) with oxcarbazepine (0.2 ‒ 2 – 20 µg/mL) and metamizol was conducted (Fig. 4). The test shows proliferation of mainly CD3 and CD4, with some proliferation of CD19 and CD56 cells, with oxcarbazepine. No proliferative response was observed with metamizol.

**Figure 4:**
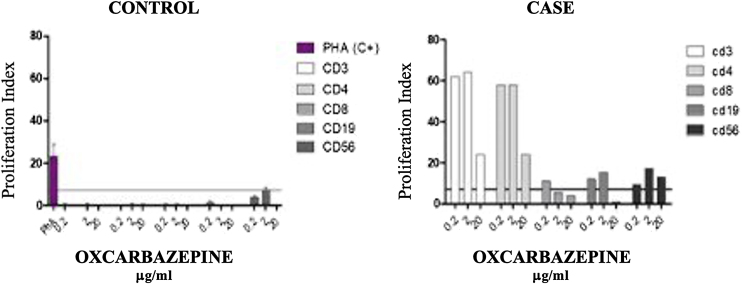
Controlled lymphocyte Transformation Test (LTT) with oxcarbazepine (0.2 ‒ 2 – 20 µg/mL). The test shows proliferation of mainly CD3 and CD4 drug speciﬁc T-cells, with some proliferation of CD19 and CD56 cells, with oxcarbazepine. The proliferation of CD56 cells suggests a cytotoxic reaction.

Oxcarbazepine-induced systemic Lupus Erythematosus was therefore diagnosed.

The rare coexistence of EM-SSJ-NET and LES lesions presents a diagnostic challenge when discussing a patient with both an infectious disease (EBV) and a pharmacological treatment (oxcarbazepine) history.

Initially, the morphology of both EM and LES lesions could be reminiscent of Rowell Syndrome (RS), but Torchia et al., who updated the RS criteria in 2012, argued for a stricter definition, restricting its use if infectious or pharmacological triggers were present.^1^

Although anti-histone antibody titration could not be performed, the normalization of complement figures, leukocytes, and ANA titers after drug suspension support the diagnosis of drug-induced lupus.^2^ The distinction between classic SSJ-NET and lupus with SSJ-NET-like lesions relies on clinical history (latency of exposure to the suspected drug), seroimmunological criteria (ANA+), and lesion distribution (photodistribution in SLE, trunk predominance in SSJ-NET).^2^, ^3^ Anatomopathological criteria can provide additional insights, although findings in these entities often overlap.^1^, ^3^

Therefore, our patient’s clinical course suggests an oxcarbazepine-induced LES with SSJ-NET-like lesions, probably precipitated by prior EBV infection.

Whereas EBV infection has already resolved, it is a known immunity disruptor, which can lead to the development of certain neoplasms, autoimmune diseases, and toxicodermas.^4^

Oxcarbazepine itself has been linked to several conditions, including SLE, erythema multiforme, SSJ, and NET. In this patient, the in vitro reactivity of Cytotoxic T Lymphocytes (CTL) to oxcarbazepine suggests it as the causal factor.^2^, ^5^

A wide variety of drugs have been associated with Drug-Induced LES (DIL). Procainamide, hydralazine, and quinidine typically produce classical DIL, which is more strongly associated with the production of anti-histone antibodies.^6^

Nevertheless, other pharmacological groups have been found linked to DIL, with lower anti-histone positiveness rates such as anticonvulsants, minocycline, propylthiouracil, and statins.^3^, ^6^, ^7^

In conclusion, while classifying patients with SSJ-NET and SLE symptoms can be challenging, its management is similar and involves the withdrawal of the triggering drug, providing supportive care, and using corticosteroids or immunosuppressants in refractory cases.

## Financial support

None declared.

## Authors' contributions

Eduardo López Vera: Contributed to the conceptualization of the case report and review; Collected and analyzed the clinical data; Conducted the literature review; and drafted the manuscript. Played a central role in coordinating the article’s development and ensuring its completion.

Elisabeth Gómez Moyano: Supervised all aspects of the study, including the conceptualization and study design. Provided critical revisions to the manuscript, ensuring its scientific accuracy and alignment with clinical guidelines. Offered guidance during the interpretation of clinical findings and literature synthesis. Approved the final version of the manuscript for submission.

María Ayala Blanca: Contributed to the review of the literature by identifying relevant references and incorporating key findings into the discussion section. Provided revisions and feedback on the clinical aspects of the manuscript, particularly related to the discussion of anatomopathological findings.

María Salas Cassinello: Contributed to the review of the literature by identifying relevant references and incorporating key findings into the discussion section. Provided revisions and feedback specifically on the laboratory findings within the clinical case.

## Conflicts of interest

None declared.

## References

[1] Torchia D., Romanelli P., Kerdel F.A. (2012). Erythema multiforme and Stevens-Johnson syndrome/toxic epidermal necrolysis associated with lupus erythematosus. J Am Acad Dermatol.

[2] Álvarez-Lario B., Bártulos-Iglesias M., Colazo-Burlato M., Macarrón-Vicente J. (2019). Carbamazepine-induced systemic lupus erythematosus: a case-based review. Eur J Rheumatol.

[3] Pretel M., Marquès L., España A. (2014). Drug-induced lupus erythematosus. Actas Dermosifiliogr.

[4] Jog N.R., James J.A. (2021). Epstein barr virus and autoimmune responses in systemic lupus erythematosus. Front Immunol.

[5] Kumkamthornkul P., Udnaen S., Tansit T., Tuchinda P., Srinoulprasert Y. (2018). Evaluation of a lymphocyte transformation test and cytokine detection assay to identify phenytoin and carbamazepine provoked DRESS or SJS/TEN in epilepsy patients. Int Immunopharmacol.

[6] Vaglio A., Grayson P.C., Fenaroli P., Gianfreda D., Boccaletti V., Ghiggeri G.M. (2018). Drug-induced lupus: traditional and new concepts. Autoimmun Rev.

[7] Alvarez-Lario B., Bartulos-Iglesias M., Colazo-Burlato M., Macarron-Vincete J. (2019). Carbamazepine-induced systemic lupus erythematosus: a case-based review. Eur J Rheumatol.

